# Sperm selection with density gradient centrifugation and swim up: effect on DNA fragmentation in viable spermatozoa

**DOI:** 10.1038/s41598-019-43981-2

**Published:** 2019-05-16

**Authors:** M. Muratori, N. Tarozzi, F. Carpentiero, S. Danti, F. M. Perrone, M. Cambi, A. Casini, C. Azzari, L. Boni, M. Maggi, A. Borini, E. Baldi

**Affiliations:** 1 Department of Experimental and Clinical Biomedical Sciences “Mario Serio”, Unit of Sexual Medicine and Andrology, Center of Excellence DeNothe, University of Florence, Florence, Italy; 2 9.baby, Family and Fertility Center, Bologna, Italy; 3 Department of Experimental and Clinical Medicine, Magna Graecia University of Catanzaro, Catanzaro, Italy; 4 Pediatric Section, Department of Health Sciences, University of Florence and Anna Meyer Children’s University Hospital, Florence, Italy; 5 Clinical Trials Center, AOU Careggi, Florence, Italy; 6 Department of Experimental and Clinical Medicine, Unit of Sexual Medicine and Andrology, Center of Excellence DeNothe, University of Florence, Florence, Italy

**Keywords:** Molecular medicine, Medical research

## Abstract

Subjects increasing sperm DNA fragmentation (sDF) during Density Gradient Centrifugation (DGC), a common sperm selection procedure in Assisted Reproduction Techniques (ARTs), experience a 50% lower probability of pregnancy. Hence, identification of these subjects is of clinical importance. Here, we investigated whether such subjects are identified with higher accuracy detecting DNA fragmentation in viable (viable sDF) instead of total spermatozoa (total sDF) and whether swim up, an alternative procedure to DGC, does not increase sDF. With DGC, we identified 10/20 subjects increasing total sDF, and 2 more subjects using viable sDF. With swim up, we identified 8/40 subjects increasing total sDF, and 8 more subjects using viable sDF. In addition, viable sDF reveals more accurately the increase of the damage when it occurs. Finally, a multivariate analysis demonstrated that the proportional increase of sDF was higher after DGC respect to swim up. In conclusion, viable sDF is a more accurate parameter to reveal the increase of the damage by selection both with swim up and DGC. Swim up increases sDF in some samples, although at a lesser extent than DGC, suggesting that it should be used to select spermatozoa for ARTs when possible.

## Introduction

Considering both primary and secondary infertility, a systematic review^1^ reported that infertility affects about 15% of couples in developed countries. In the last decades, Assisted Reproductive Technologies (ARTs) offered many infertile couples the opportunity to have a child. However, these technologies are, at best, 30% effective in terms of live birth^2^ and, consequently, many couples experience a failure or need several attempts to achieve parenthood. The infertility background of the couple can account for this high rate of failure^3^, but also the *in vitro* conditions, far from the natural ones, concur to decrease the success of ARTs. *In vitro* manipulation of gametes and embryo can expose them to several noxious conditions that are present in a typical clinical ART setting^4^. In natural conditions, spermatozoa undergo an intensive quantitative and qualitative selection process, likely assuring that only the best gametes reach the oocyte^5^. In the ART setting, the most popular procedure to select spermatozoa is the Density Gradient Centrifugation (DGC), where gametes are forced to cross a gradient made of colloidal silicon and are separated based on their density. DGC yields sperm populations with higher motility^6^, better morphology and maturity^7^ respect to whole semen. However, recent evidence indicates that DGC may increase sperm DNA fragmentation (sDF) levels^8,9^, a parameter that negatively impacts reproductive outcomes after ARTs^10–14^. In particular, our group reported that DGC increases sDF in about 50% of infertile couples treated by IVF/ICSI^9^. More importantly, these subjects experienced a 50% lower pregnancy rate with respect to those showing a decrease of DNA damage during selection^9^. In this scenario, it appears important to identify the subjects increasing sDF during selection with DGC as well as to determine whether alternative sperm selection procedures, do not increase sDF. An alternative sperm selection procedure used in ART settings is swim up. At present there are no studies indicating an increase of sDF during selection with swim up, however, these studies (for review see^15^) only report the average values of total sDF before and after selection without considering individual samples.

As mentioned, selection procedures highly ameliorate sperm motility and eliminate poorly motile and/or dead spermatozoa, the latter being mostly DNA fragmented^16^. In such a situation, the comparison of sDF in samples before and after DGC is performed in different sperm populations, the first formed by dead and viable and the second by virtually only viable spermatozoa. In addition, when calculating the difference between sDF of total spermatozoa (total sDF) in samples before and after selection, the elimination of dead, DNA fragmented spermatozoa during selection could partially mask the eventual increase of DNA damage in the viable cells. Conversely, detecting sDF in the viable fraction (viable sDF) of both the pre- and the post-selection sperm populations is not affected by deletion of dead spermatozoa. Although also this strategy compares different pre- and post-selection populations (the post-selection representing only part of the viable pre-selection one) it should be more accurate to reveal the increase of the damage and the actual number of subjects increasing sDF during selection.

The aim of the present study was to evaluate sDF after selection procedures (DGC and swim up) by using a novel technique able to reveal sDF simultaneously in the viable and in the total sperm population and by considering individual samples. In addition, we aimed to verify whether viable unmasks subjects undergoing sperm DNA damage during selection with respect to total sDF.

## Material and Methods

### Patients and sample collection and preparation

Semen samples were consecutively collected from male partners of infertile couples who presented at Tecnobios Procreazione (Bologna, Italy) from January to December 2017 for routine semen analysis and diagnostic tests of function. Semen samples were collected by masturbation after 2–7 days abstinence period and analyzed for sperm number, concentration, motility and morphology according to WHO procedures^17^. Briefly, spermatozoa were counted by improved Neubauer hemocytometer after dilution with fixative, whereas motility and morphology (after Diff-Quick staining) were evaluated by optical microscopy scoring 200 spermatozoa. Samples with leucocytospermia or semen bacteria were excluded by the study. Diagnostic tests of function were conducted by preparing spermatozoa with DGC (n = 20, age: 39.6 ± 4.1, range: 31–47) or swim up (n = 20, age: 36.0 ± 6.6, range: 28–48) depending on the quality of basal semen, in particular, swim up was chosen in case of good sperm number and motility^17^. These swim up selected samples with good basal semen quality are indicated as SW-G.

Swim up was also performed in samples collected from 25 subjects selected using the following inclusion criteria: 20.0% < progressive motility < 59.1% or 12.4 million/ml < sperm concentration < 104.9 million/ml or both. 20% motility and 12.4 million/ml are the minimum values of, respectively, sperm progressive motility and concentration whereas 59.1% and 104.9 million/ml are the mean + SD of the same parameters in the 20 samples processed by DGC. Only 20 subjects (age: 36.0 ± 9.3, range: 24–60) were included in the study as in the remaining 5 we could not obtain a sufficient number of spermatozoa for labelling sperm DNA breaks. These swim up selected samples are indicated as SW-P.

DGC was performed by discontinuous PureSperm (Nidacon, Gothenburg, Sweden) gradient. Briefly, semen samples were layered upon a 40:80% PureSperm density gradient and processed by centrifuge at 600 × g for 15 min. The recovered 80% pellet was resuspended in 1 ml of PureSperm Wash medium (Nidacon, Gothemberg, Sweden), centrifuged for 10 min at 200 × g to eliminate remaining colloidal particles, and finally resuspended in 1 ml of sperm culture medium.

Swim up selection was performed according to the clinical practice at Tecnobios Riproduzione and most ART Laboratories in Italy^18–22^. Briefly, a volume of PureSperm Wash medium/10% Human Serum Albumin was added to an identical volume of semen and then centrifuged at 400 × g for 15 minutes. The supernatant was discarded and the pellet was suspended in pre-warmed 300 µl of the culture medium and then gently over-layered with 1.0 ml of the same medium. The sample was then kept at 37 °C for 45 min in 5% CO2. One ml of supernatant containing actively motile spermatozoa was then removed and placed in a different tube.

After sperm selection, with DGC or swim up, evaluation of concentration and progressive motility was repeated. Then 2–10 × 10^6^ spermatozoa were processed to detect sDF in total and viable sperm fraction (see below).

The study was approved by the internal institutional review board of Tecnobios Riproduzione (Bologna, Italy). Informed consent was obtained by recruited subjects. All the methods used in this study were carried out in accordance with the approved guidelines.

### LiveTUNEL

For detection of sDF both in total and viable sperm fractions, we coupled TUNEL (terminal deoxynucleotidyl transferase (TdT)-mediated fluorescein-dUTP nick end labeling) with the staining of dead cells with LIVE⁄ DEAD Fixable Far Red Dead Cell Stain Kit (L10120, Life Technologies, Paisley, UK) (from herein indicated as LiveTUNEL). L10120 is able to bind dead cells and the labelling is stably kept in the cells after sample fixation and permeabilization^23^. After washing with HTF medium, fresh semen samples were incubated for 1 h at RT, in the dark, in 500 μl of phosphate buffered saline (PBS) with L10120 (diluted 1:10 000). After further washing, the samples were fixed by 500 μL of 4% paraformaldehyde in PBS, pH 7.4, for 30 min at RT. Fixed samples were then labelled by TUNEL as described elsewhere^24^. Briefly, sperm were washed twice by PBS/1% Bovine Serum Albumine and permeabilized with 0.1% Triton X-100 in 100 µL of 0.1% sodium citrate for 4 min in ice. After washing two times, the labelling reaction was performed by incubating sperm in 50 µL of labelling solution (supplied with the *In Situ* Cell Death Detection Kit, fluorescein, Roche Molecular Biochemicals, Milan, Italy) containing the TdT enzyme, for 1 hour at 37 °C in the dark. Finally, samples were washed twice, re-suspended in PBS and stained with DAPI (1 μg/ml) for 15 min in the dark at RT until acquisition with flow cytometry. For each test sample, a negative control was also prepared by omitting TdT.

### Flow cytometric analyses

Flow cytometric analyses were conducted by a FACSAria II flow cytometer (BD Biosciences, Franklin Lakes, NJ, USA) equipped with a violet laser, a blue laser and a red laser for excitation, respectively, at 405 nm, 488 nm and 633 nm. Before acquiring, spermatozoa were filtered by 50 μm Syringe Filcons (BD Biosciences, Franklin Lakes, NJ, USA). Blu (DAPI), green (FITC), and far red (L10120) fluorescence was revealed by PTMs equipped with, respectively, 450/40, 530/30, and 660/20 BP filters.

**Figure 1:**
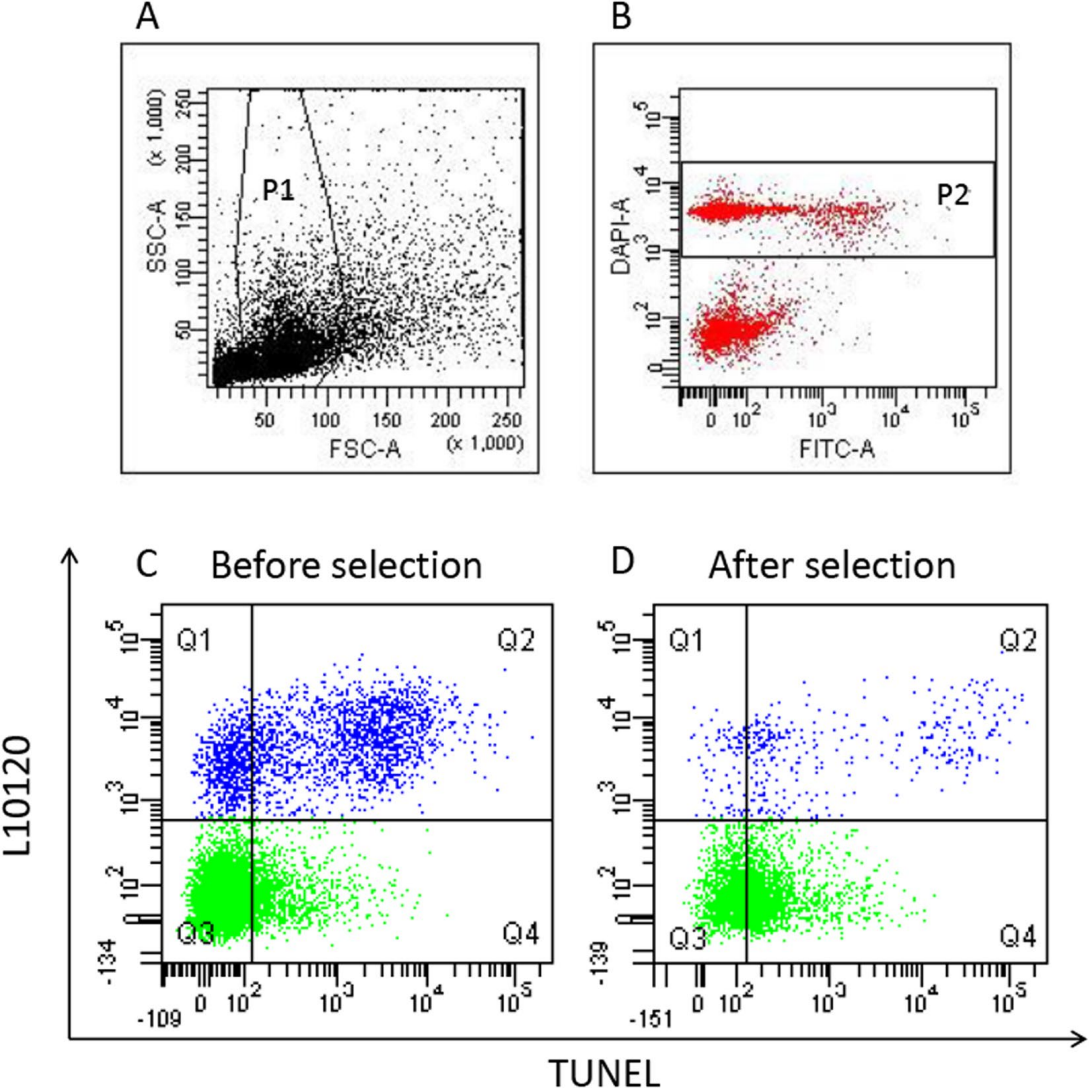
LiveTUNEL. Typical dot plots of LiveTUNEL. In the flame shaped region of the FSC/SSC dot plot (P1) (**A**), spermatozoa are gated as DAPI positive events (P2) (**B**). L10120/TUNEL dot plots depict spermatozoa by distinguishing between viable (green) and dead (blue) cells in a sample before (**C**) and after (**D**) DGC selection. Q1 quadrant: L10120+/TUNEL− events (dead, not DNA fragmented spermatozoa); Q2 quadrant: L10120+/TUNEL+ (dead, DNA fragmented spermatozoa); Q3 quadrant: L10120−/TUNEL− (viable, not DNA fragmented spermatozoa); Q4 quadrant: L10120−/TUNEL+ (viable, DNA fragmented spermatozoa).

BD FACSdiva Software (BD Biosciences, Franklin Lakes, NJ, USA) was used for acquisition and data analysis. Spermatozoa were gated by the characteristic FSC/SSC flame shaped region (P1) and, within it, by the region containing DAPI labelled events (P2) (Fig. 1A,B). Within P2, a further gate was drawn for viable sperm. For each sample, 8000 viable sperm (L10120 unstained events, Fig. 1C,D, Q3 + Q4 quadrants) were recorded. For data analysis, in the L10120/TUNEL dot plot of the negative controls, quadrants were set to include about 99% of the dead and the viable sperm and then copied in the corresponding test samples where the DNA fragmented dead and viable spermatozoa are located in the Q2 and Q4 quadrants, respectively (Fig. 1C,D). Total SDF was calculated as percentage of the events in the Q2 and Q4 quadrants on total sperm population; viable sDF was calculated as percentage of the events in the Q4 quadrant on viable sperm population (events in Q3 and Q4 quadrants) (Fig. 1C,D).

### Statistical analysis

Results are expressed as mean ± SD or mean ± SE. The unpaired and paired Student’s t test and analysis of variance (ANOVA) were used to assess statistically significant differences between the compared groups. Bivariate correlation between the variation of total sDF and the dead sperm deletion during selection was evaluated by calculating the Spearman’s correlation coefficient (r). Total and viable sDF were considered increased or decreased after selection when the difference between the value before and after selection was, respectively, negative or positive. The before-after univariate and multivariate modeling was performed according to the analyses of covariance (ANCOVA) with Tukey-Kramer adjustment for multiple comparisons when required. Proportional change was calculated as: (average value after selection - average value before selection)/average value before selection. A p-value of 0.05 was considered as statistically significant. Statistical analyses were carried out using Microcal Origin software, 6.1 version (MicroCal Software Inc., Northampton, MA, USA), SPSS software, 25 version, for Windows (SPSS, Inc., Chicago, IL, USA) and SAS Statistical Software, version 9.2 (SAS Institute Inc, Cary, NC).

## Results

### LiveTUNEL

To evaluate the effect of selection on sDF levels, we used a flow cytometric technique (LiveTUNEL) able to detect simultaneously total and viable sDF (Fig. 1, see materials and methods for the strategy of calculation of the two parameters). In the unselected samples most of the DNA fragmented spermatozoa are dead (Fig. 1C, Q1 and Q2 quadrants)^15^ and thus mostly deleted during selection (Fig. 1D).

### Total and viable sDF levels before and after DGC

**Figure 2:**
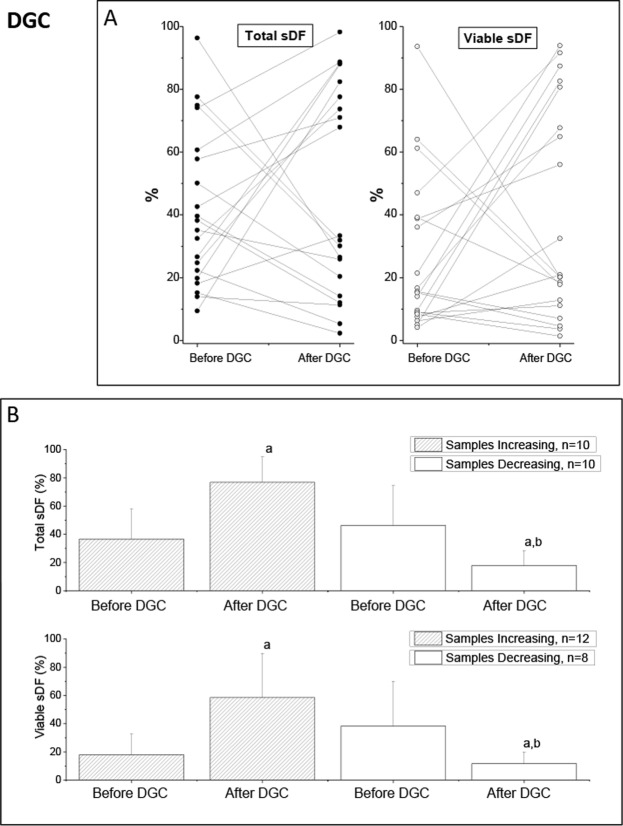
Effect of DGC on total and viable sDF. (**A**) Values of total and viable sDF before and after selection by DGC in 20 individual semen samples. (**B)** Average values of total (upper graphs) and viable sDF (lower graphs) in samples increasing and decreasing sDF after selection. a = statistically significant difference between values before and after DGC (t-Test, paired data), p < 0.001; b = statistically significant difference between samples increasing and samples decreasing sDF, as assessed after DGC (ANCOVA, after adjustment for basal parameters), p < 0.001.

Table 1 (DGC) reports the average values of conventional semen parameters before selection and of progressive motility after selection of 20 subjects processed with DGC, whereas Table 2 (DGC) shows the average total and viable sDF values before and after DGC in the same subjects. As shown, we found no difference in average sDF levels before and after selection, at variance with sperm motility that highly increased (Table 1, DGC). In agreement with previous results^9^, when we analyzed the individual subjects, we found both samples increasing and decreasing sDF in the total (Fig. 2A, left panel) and in the viable (Fig. 2A, right panel) populations. Average pre-selection levels of total and viable sDF were not different (p = 0.398 and p = 0.068, respectively) in samples increasing and decreasing the damage during selection (Fig. 2B). Similarly, conventional semen parameters, post-selection sperm progressive motility as well as the improvement of progressive motility (Supplemental Tables 1 and 2, DGC) did not differ between subjects increasing and decreasing sDF during selection. Interestingly, we found 10 (50%) subjects increasing total and 2 more (n = 12, 60%) increasing the viable sDF. The proportional increase of sDF in the 12 samples increasing viable sDF, was 2.26 (95%CI:1.52–3.00) for viable and 0.89 (95%CI:0.47–1.31) for total population. The proportional decreases in viable (−0.69, 95%CI: −0.98–0.40) and total (−0.62,95%CI: −0.80–0.44) sDF were similar in the 8 samples decreasing viable sDF.

**Table 1:** Semen parameters and sDF in the recruited subjects for DGC, SW-G and SW-P selection.

Variable	DGC (n = 20)	SW-G (n = 20)	SW-P (n = 20)	P-value^a^	P-value^b^	P-value^c^
Sperm Concentration (millions/ml)	65.5 ± 39.4	137.3 ± 106.2	79.6 ± 57.1	0.011	0.372	0.055
Sperm Number (millions/ejaculate)	215.5 ± 162.0	453.2 ± 330.3	293.8 ± 253.3	0.010	0.252	0.136
Progressive Motility before selection (%)	45.6 ± 13.5	54.8 ± 9.4	52.4 ± 10.7	0.018	0.083	0.552
Progressive Motility after selection (%)	78.8 ± 8.0	84.8 ± 13.6	81.0 ± 9.3	0.101	0.429	0.314
P-value^d^	0.001	0.001	0.001			

Data are mean ± SD. ^a^DGC vs SW-G; ^b^DGC vs SW-P; ^c^SW-G vs SW-P, t-Test, independent data. ^d^Before versus after selection; t-Test, paired data. DGC, Density Gradient Centrifugation. SW-G, swim up conducted with good quality semen samples. SW-P, swim up conducted with poor quality semen samples.

**Table 2:** Semen parameters and sDF in the recruited subjects for DGC, SW-G and SW-P selection.

Variable	DGC (n = 20)	SW-G (n = 20)	SW-P (n = 20)
Total sDF before selection (%)	41.5 ± 24.9	41.5 ± 19.5	41.5 ± 12.1
Total sDF after selection (%)	47.4 ± 33.4	27.0 ± 23.4	31.4 ± 21.0
P-value^a^	0.522	0.001	0.080
Viable sDF before selection (%)	26.1 ± 24.5	24.8 ± 20.4	20.5 ± 13.0
Viable sDF after selection (%)	39.8 ± 33.9	23.6 ± 22.0	21.7 ± 21.4
P-value^a^	0.167	0.707	p = 0.825

Data are mean ± SD. ^a^Before versus after selection; t-Test, paired data. DGC, Density Gradient Centrifugation. SW-G, swim up conducted with good quality semen samples. SW-P, swim up conducted with poor quality semen samples. sDF, sperm DNA fragmentation

### sDF levels in total and viable sperm populations before and after swim up

In diagnostic workup before ARTs, the choice of selection procedure (DGC or swim up) is based on WHO manual^17^, thus, swim up is usually chosen for semen samples with better quality. Here, we performed swim up in 20 semen samples from subjects with good semen parameters (SW-G) (Table 1) and in 20 semen samples from subjects with basal semen parameters that normally would be processed with DGC in ART laboratories (SW-P) (Table 1).

**Figure 3:**
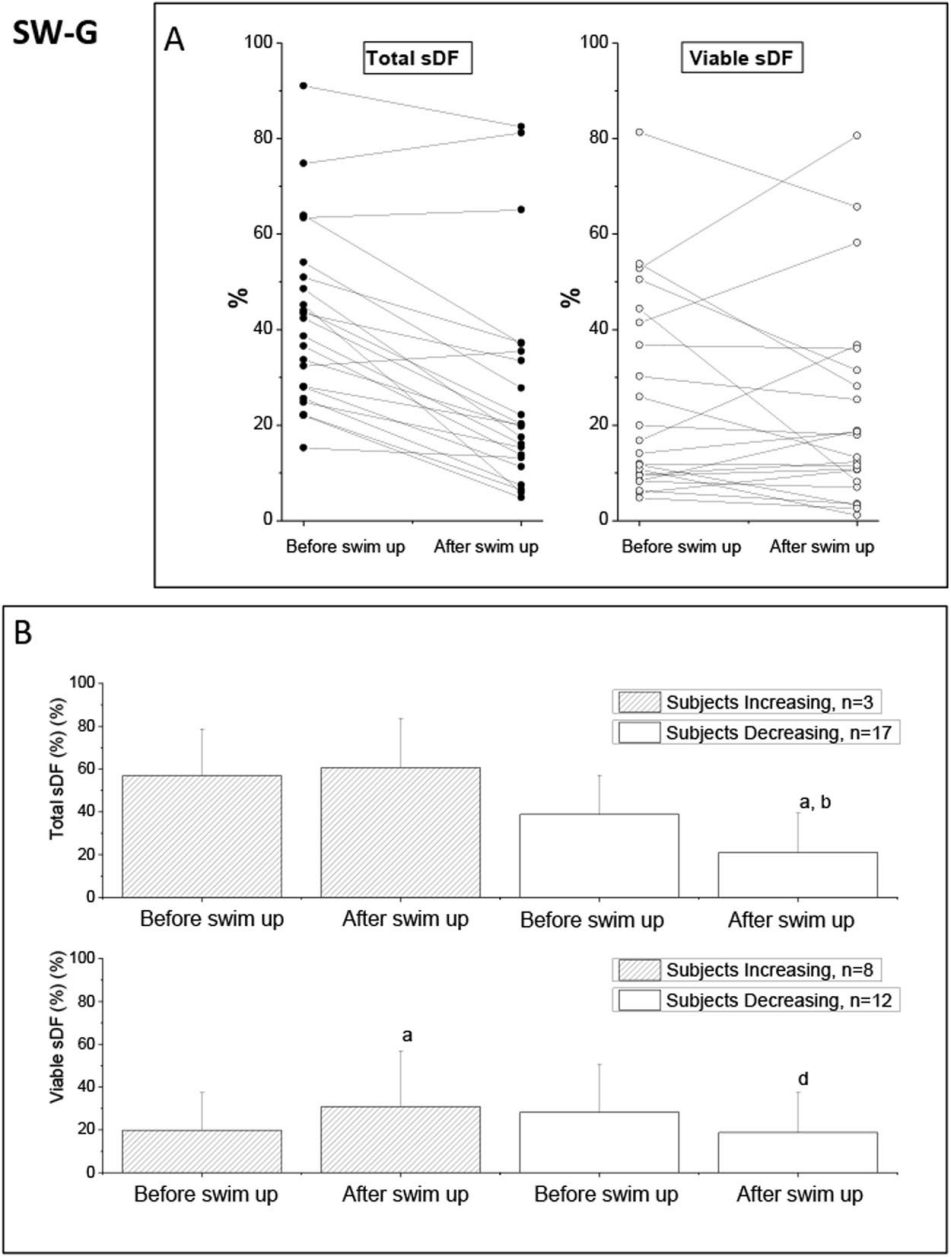
Effect of SWIM UP on total and viable sDF in SW-G samples. (**A**) Values of total and viable sDF before and after selection by swim up in the 20 individual SW-G samples. (**B**) Average values of total (upper graphs) and viable sDF (lower graphs) in samples increasing and decreasing sDF after selection. a = statistically significant difference between values before and after swim up (t-Test, paired data), p < 0.001, <0.05, and <0.01, respectively, for samples decreasing total sDF, sample increasing and decreasing viable sDF; b = statistically significant difference between samples increasing and samples decreasing sDF, as assessed after swim up (ANCOVA, after adjustment for basal parameters), p < 0.01.

Table 1 (SW-G) reports the average values of conventional semen parameters before selection and of progressive motility after selection of the 20 SW-G subjects processed with swim up, whereas Table 2 (SW-G) shows the average values of total and viable sDF before and after swim up in the same subjects. The average value of total sDF decreased significantly after selection, whereas it did not change when calculated in the viable sperm population (Table 2, SW-G). When we analyzed individual subjects, we found both samples increasing and decreasing the damage, when considering both total (Fig. 3A, left panel) and viable (Fig. 3A, right panel) sDF, despite the expected increase of motility in both (Supplemental Tables 1 and 2, SW-G). Figure 3B reports the average values of total (upper panel) and viable (lower panel) sDF before and after swim up, in SW-G samples increasing and decreasing the damage during selection. No differences were observed in the basal values of sDF (total and viable, p = 0.143 and p = 0.482, respectively, Fig. 3B), conventional semen parameters, progressive motility after selection, and improvement of progressive motility (Supplemental Tables 1 and 2, SW-G). For SW-G samples, the number of those showing an increase in sDF was 8 (40%) when calculated in the viable population, 5 more of the 3 (15%) observed with total population. In the 8 samples showing an increase in viable sDF, the proportional increase of the damage was 0.56 (95%CI: −0.26–1.38) for viable and −0.15 (95%CI: −0.62–0.31) for total population, whereas proportional decreases in the remaining 12 samples were of −0.45 (95%CI: −0.64–0.29) and −0.33 (95%CI: −0.66–0.01) in total and viable sDF, respectively.

**Figure 4:**
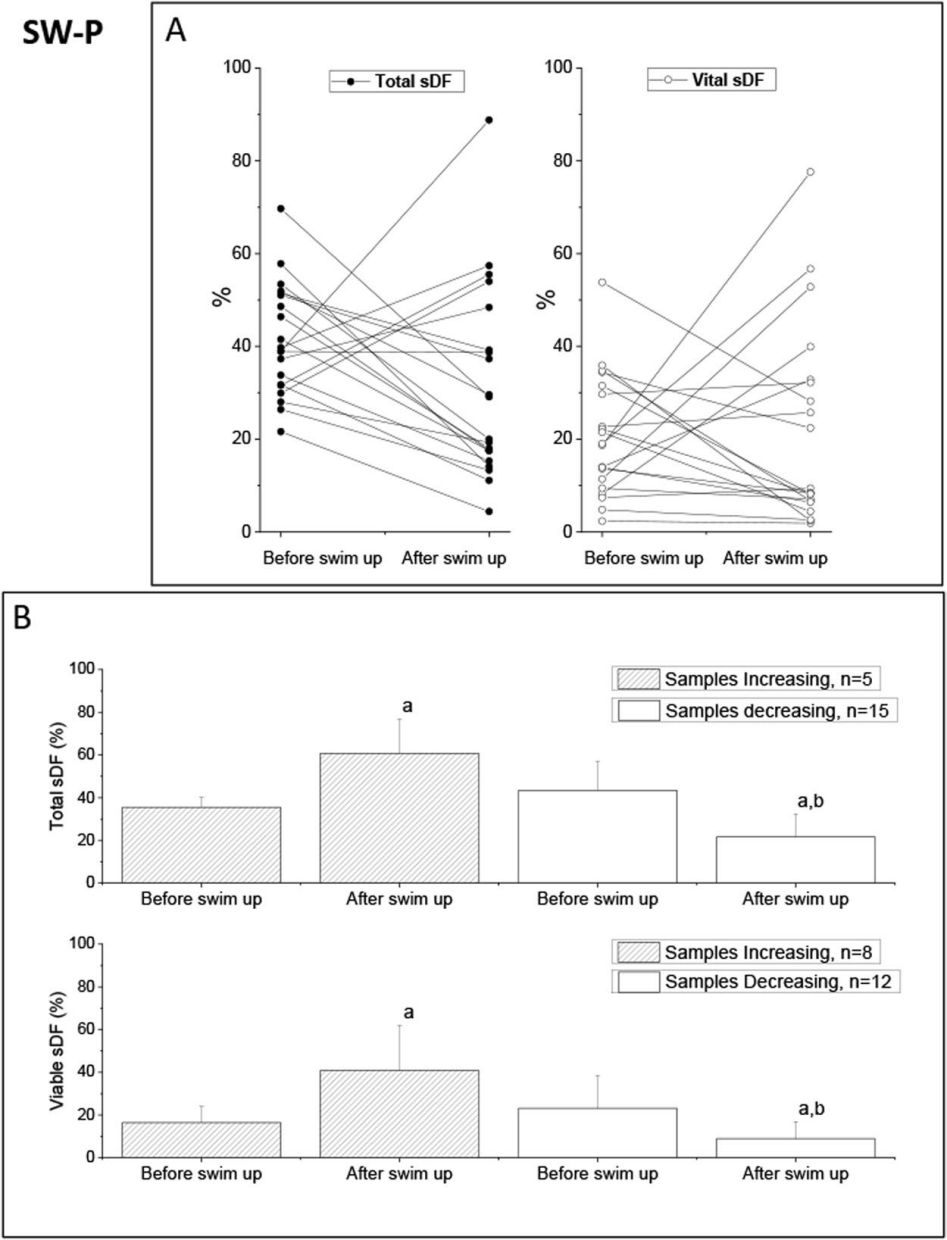
Effect of swim up on total and viable sDF in SW-P samples. (**A**) Values of total and viable sDF before and after selection by swim up in the 20 individual SW-P semen samples. (**B**) Average values of total (upper graphs) and viable sDF (lower graphs) in samples increasing and decreasing sDF after selection. a = statistically significant difference between values before and after swim up (t-Test, paired data), p < 0.05 and p < 0.001, respectively, in samples increasing and decreasing total sDF; p < 0.05, and p < 0.01, respectively, in samples increasing and decreasing viable sDF; b = statistically significant difference between samples increasing and samples decreasing sDF, as assessed after swim up (ANCOVA, after adjusting for basal parameters), p < 0.001.

In the SW-P samples, there was no difference in the average total and viable sDF after selection, at variance with motility that highly increased (Tables 1 and 2, SW-P). The individual analysis showed the presence of samples increasing and decreasing DNA damage after selection both when calculated in total (Fig. 4A, left panel) and viable (Fig. 4A, right panel) sperm population. The average (total, upper panels and viable, lower panels) sDF levels before and after swim up, in SW-P samples increasing and decreasing the damage during selection, are shown in Fig. 4B. As can be observed, basal values of total and viable sDF were not different (p = 0.212 and p = 0.263, respectively, Fig. 4B). Similarly, no difference was observed in progressive motility and in the improvement of the parameter with selection (Supplemental Tables 1 and 2). Samples showing an increase in total sDF had a lower sperm number and concentration than samples showing a decrease (Supplemental Table 1, SW-P) whereas such differences were not significant when viable sDF was considered (Supplemental Tables 2, SW-P). The number of samples showing an increase in viable was 8 (40%), 3 more respect to total sDF (n = 5, 25%). In the 8 samples with increase in viable sDF, the proportional increase of the damage was 1.49 (95%CI:0.50–2.49) for viable and 0.24 (95%CI: −0.21–0.71) for total sDF. Similar decrease of total (−0.54,95%CI: −0.71–0.36) and viable (−0.61,95%CI: −1.01–0.22) sDF was found in the remaining 12 samples.

Overall, our results suggest that LiveTUNEL unmasks some subjects undergoing DNA damage during selection respect to conventional methods revealing total sDF. To verify further whether elimination of dead sperm during selection affects the difference of total sDF before and after selection, we plotted such difference against the percentage of dead sperm eliminated during selection in the 60 samples (Supplemental Fig. 1). We found that the higher was the decrease of dead sperm, the higher was the difference of total sDF before and after selection (r = 0.413, p = 0.001, n = 60).

### Post-selection sDF levels in viable and total populations

**Figure 5:**
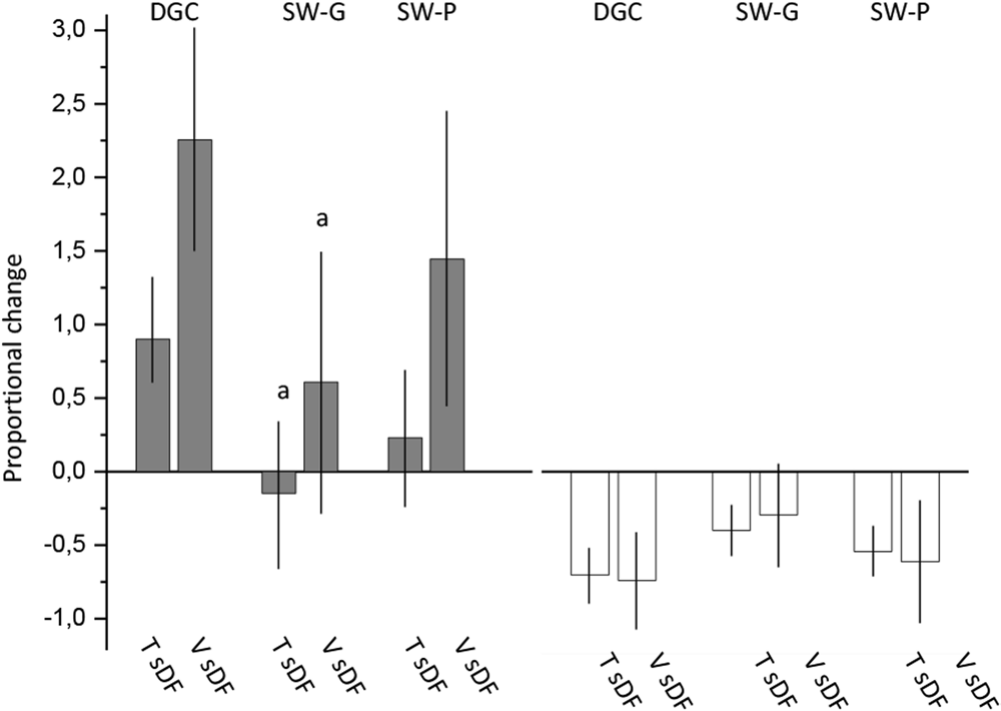
Comparison between DGC, SW-G and SW-P in subjects increasing or decreasing viable sDF. Proportional change of total and viable sDF in subjects increasing (grey columns) and decreasing (white columns) viable sDF after selection with DGC and swim up in the SW-G and SW-P samples, after adjustment for basal progressive motility and sperm number. a = statistically significant difference in the increases of total and viable sDF between DGC and SW-G (ANCOVA), p < 0.05. T sDF, Total sDF; V sDF, Viable sDF.

To compare the effect on sDF levels after DGC and swim up procedures, we analysed the differences in post-selection sDF levels among the three experimental sets with a multivariate analysis (ANCOVA), after adjustment for basal sperm parameters. As shown in Table 3, although the difference in total sDF between DGC and SW-G in unadjusted or adjusted model for basal sDF levels was significant, such significance was lost with adjustment for basal sperm progressive motility and number. No difference was observed for post-selection viable sDF levels in the three experimental sets with any of the three statistical models (Table 3). Unadjusted and adjusted values of total and viable sDF after selection in the three experimental sets are reported in Supplemental Table 3. Next, we considered only the samples showing an increase in viable sDF, to evaluate whether the proportional increase of sDF was different among the three experimental groups. After adjustment for basal progressive motility and sperm number, we found that the increase of both total (p = 0.018) and viable sDF (p = 0.047) in samples processed by DGC was higher respect to SW-G but not SW-P samples (p = 0.139 and p = 0.247 for total and viable sDF respectively) (Fig. 5). No difference was found between SW-G and SW-P (p = 0.521 and p = 0.606 for total and viable sDF respectively). Figure 5 also shows the proportional decrease of total and viable sDF in samples decreasing the damage in the three experimental groups, after adjustment.

**Table 3:** Differences between the selection procedures in total and viable sDF values as obtained after selection, using a multivariate model.

	Overall	DGC vs SW-G		DGC vs SW-P		SW-G vs SW-P		SWIMUP vs SWIMUP(SS)
	p value	d (95% CI)	P value	d (95% CI)	P value	d (95% CI)	P value	
**Total sDF after selection**								
Unadjusted	0.044	20.45 0.28, 40.63)	0.046	16.02 (−4.16,36.20)	0.144	−4.43 (−24.61.15.74)	0.857	
Model 1	0.037	20.46 (0.89,40.02)	0.038	16.02 (−3.54,35.58)	0.129	−4.44 (−24.00,15.13)	0.849	
Model 2	0.199	13.32 (−7.90,34.53)	0.293	13.68 (−6.03,33.39)	0.225	0.36 (−19.29,20.02)	0.999	
**Viable sDF after selection**								
Unadjusted	0.067	16.18 (−3.92,36.28)	0.138	18.07 (−2.03,38.17)	0.086	1.89 (−18.20,21.99)	0.972	
Model 1	0.087	15.85 (−4.03,35.73)	0.142	16.56 (−3.44,36.57)	0.123	0.71 (−19.24,20.67)	0.996	
Model 2	0.213	9.44 (−12.12,31.01)	0.546	14.88 (−5.27,35.04)	0.186	5.44 (−14.65,25.52)	0.792	

d = difference.

Model 1, adjustment for basal value of sDF (total or viable).

Model 2, adjustment for basal value of sDF (total and viable) and for basal sperm progressive motility and number. DGC, density gradient centrifugation. SW-G, swim up conducted with good quality semen samples. SW-P, swim up conducted with poor quality semen samples. sDF, sperm DNA fragmentation

## Discussion

Ours is the first study evaluating pre- and post- selection sDF values simultaneously in the total and viable sperm populations after both DGC and swim up considering single samples, thus allowing the identification of those subjects increasing the damage after selection. We confirm^9^ that among sub/infertile men there is a fraction of subjects undergoing an increase of sperm DNA damage during selection with DGC and extend this result also to swim up, an alternative selection procedure often used in ART laboratories. This result is important in view of the lower pregnancy rate after IVF/ICSI treatment in subjects increasing DNA damage during selection^9^. In addition, we show here that the effect of sperm selection with DGC and swim up on sDF levels can be unmasked in some samples by distinguishing viable and dead spermatozoa.

In this study, we detected sDF by LiveTUNEL, a novel flow cytometric technique revealing the DNA damage both in the total and in the viable cells. At variance with previous similar techniques^16,25^, LiveTUNEL excludes, beside germ cells and leukocytes, also semen apoptotic bodies, that affect the measures of total and viable sDF, as extensively discussed elsewhere^26^. The exclusion of apoptotic bodies and of dead spermatozoa in the calculation of viable sDF, can explain the much higher percentages of basal viable DNA fragmented spermatozoa (24.0 ± 19.7%, n = 60) found here with respect to previous studies (below 5% in^16^ and^25^). It is also possible that semen samples used in previous studies^16,25^ had better sperm parameters which might be associated to a lower sDF in live spermatozoa. As apoptotic bodies are deleted during selection^27^, it is anticipated that they differently affect the sDF measures in the unselected and in the selected samples and thus bias the results of the effect of selection on sperm DNA.

**Figure 6:**
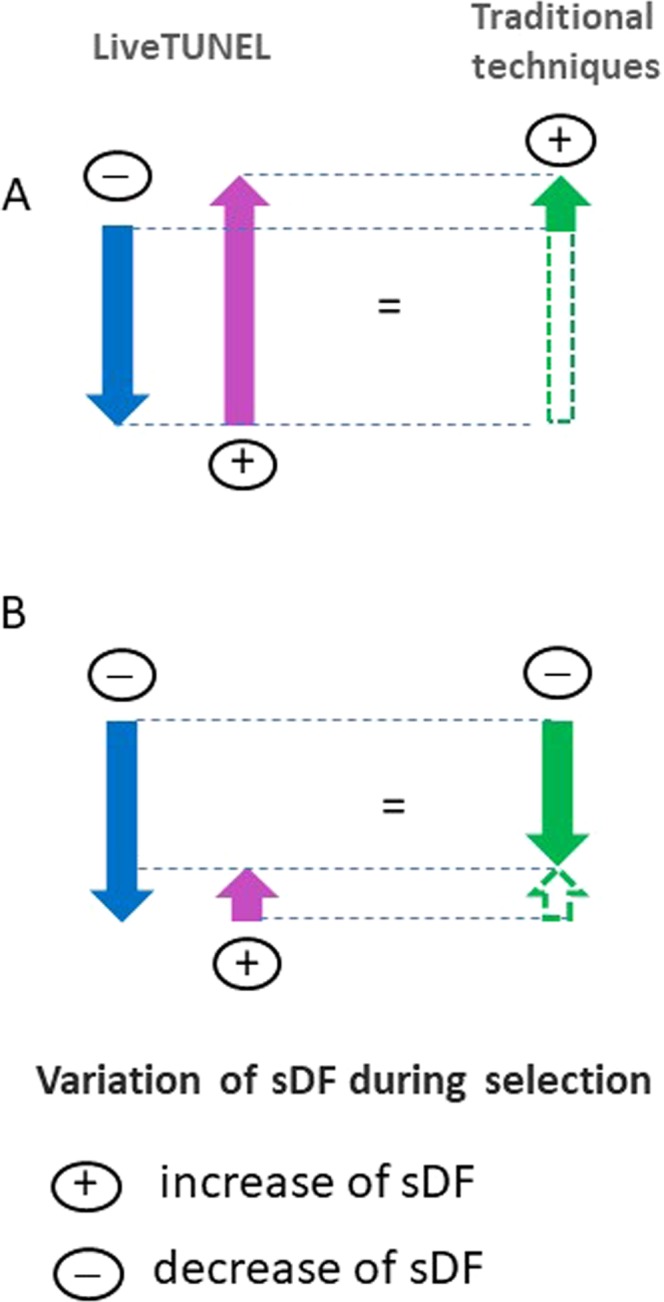
Variation of sDF during selection as evaluated by traditional techniques (revealing total sDF) and liveTUNEL (revealing viable sDF). LiveTUNEL unmasks samples increasing sDF respect to traditional techniques. (**A**) A sample with an increase of viable sDF (pink arrow) which is higher than the decrease due to deletion of DNA fragmented dead spermatozoa (blue arrow). In this case, both traditional techniques (total sDF, green arrow) and LiveTUNEL (viable sDF, pink arrow) can identify the increasing sample. (**B**) A sample with an increase of viable sDF (pink arrow) which is lower than the decrease due to deletion of DNA fragmented dead spermatozoa (blue arrow). In this case only LiveTUNEL (viable sDF, pink arrow) can identify the increasing sample. Note also that traditional techniques reveal only a part of increase (closed green arrow in A). Conversely, LiveTUNEL (pink arrow) detects all the increase. Dashed lines represent the amount of increase of sDF not detected by traditional techniques.

The use of LiveTUNEL allowed us to compare sDF values before and after selection in the population of viable spermatozoa. Since sperm populations before and after selection are differently composed (due to elimination of most dead spermatozoa) we reasoned that the comparison is more accurate evaluating viable than total sDF as detected with traditional techniques (Fig. 6). Indeed, viable sDF not only unmasks some subjects increasing sDF levels both after DGC and swim up, but also reveals more accurately the amount of the increase of the damage when it occurs (Fig. 6). The finding that the variations of total sDF strictly correlates to the deletion of dead spermatozoa (Supplemental Fig. 1), further supports LiveTUNEL as a more accurate method to evaluate the effect of selection on sperm DNA quality, independent from the negative selection of dead cells.

Another important aspect of evaluating viable SDF is that the damage is determined in the sperm population expected to impact most the reproductive outcomes, as DNA fragmented viable spermatozoa may have the capability to reach and fertilize the oocyte^28^.

Besides the use of traditional techniques to evaluate sDF, the presence of subjects increasing or decreasing DNA damage during selection has been so far neglected also because, in many studies, data were presented as average values before and after selection. Such studies often reported no or scarce variation of the damage after swim up^29,30^ but, above all, DGC^31–33^. Our results indicate that future studies on this topic not only should focus on DNA fragmentation in the viable sperm fraction, but also report pre- and post-selection individual values.

Regarding the mechanisms provoking the increase of sDF during selection in some subjects, an induction of a de novo DNA damage appears to be the most probable hypothesis, although our experimental design does not allow to exclude a positive selection of DNA fragmented spermatozoa by DGC and swim up. It is also possible that the increase of DNA damage after selection might be a consequence of the “tip of the iceberg” effect^34^, i.e. the existence of a basal DNA damage below the threshold of detection, which could be unmasked by the insult of selection. This interpretation would completely change the meaning of selection induced DNA damage: not a de novo DNA damage, but unmasked, “latent” DNA damage^35^.

The nature of the putative insult inducing DNA damage is not clear. Heavy metal contamination of the colloidal silicon gradients has been reported to induce a localized oxidative attack in turn promoting DNA breaks^8^ in samples selected by DGC. Surprisingly, also swim up procedure, which does not use gradients, increased DNA damage in some subjects and thus alternative sources of oxidative injury appear to be involved. Among these, centrifugation steps^4^, exposure to visible light^36^, and handling under atmospheric oxygen concentration^37^ can be hypothesized. Contamination by heavy metals of the culture media used for swim up cannot be excluded as well.

Whatever is the nature of the insult producing the damage, the outcome of selection seems to depend also on unknown vulnerable sperm traits present in subjects increasing the damage, as not all the samples undergo it. Such traits could be due to defects in the ability to protect DNA, including alteration of the proper chromatin protamination and/or maturation or ineffective or decreased antioxidant cell defenses. Intriguingly, subjects increasing and decreasing DNA damage during selection did not differ for conventional semen parameters as well as the value of sDF before selection (Figs 2–4, panels B), suggesting that these features of vulnerability do not affect the basal DNA damage, contrary to what expected. We cannot exclude the effect of other individual characteristics (varicocele, obesity, smoking and other possible confounders) as these data were not available in our study. However, in our previous study^9^ male factor was not different between subjects increasing and decreasing sDF after selection with DGC.

One limitation of the present study is the lack of a comparison between DGC and swim up in the same subjects. This strategy, however, can be pursued only in subjects showing a good semen quality (in order to have a sufficient number of spermatozoa for labeling with liveTUNEL the pre-and post-selection sperm populations processed by both selection procedures). Conversely, several subjects undergoing ARTs show a poor to moderate semen quality. Our experimental design allowed us to include a high number of subjects with low semen quality. In an attempt to overcome this limitation, we statistically compared the two procedures using a multivariate analysis adjusting for basal values of sDF, progressive motility and sperm number. We found no difference between DGC and swim up (both SW-G and SW-P), albeit a tendency to higher sDF values after DGC than after swim up was evident. With a similar multivariate analysis, we also compared, in subjects increasing the damage, the proportional increase of sDF with the two procedures. A difference was found between DGC and SW-G but not SW-P, although the proportional change of DNA damage was not statistically different between the two groups of samples processed by swim up and the proportional increase in SW-P tend to be much lower respect to DGC.

In a previous study, our group demonstrated that the increase of sDF during DGC selection provoked a decrease in pregnancy rate in couples treated by IVF/ICSI^9^. Based on results of the present study, preparing spermatozoa with swim up might improve the reproductive success with respect to DGC at least for subjects with good semen parameters. Although Hammadeh *et al*.^38^ failed to find significant differences in the ART outcomes with spermatozoa prepared by swim up respect to DGC, a recent large study reported opposite results^39^. Further large-scale studies will be necessary to definitively clarify this issue^40^.

In conclusion, viable sDF is a more suitable parameter to assess the effect of selection on sperm DNA quality. DGC and swim up increase sDF in viable spermatozoa in, respectively, about 60% and 40% of the subjects. Furthermore, the increase of the damage is lower in semen samples with good parameters processed with swim up, thus suggesting a preferential use of the latter procedure to select spermatozoa for ARTs.

## Supplementary information

Supplemental Information

## Data Availability

The datasets generated and/or analysed during the current study are available from the corresponding author on reasonable request.
