# Supplementary material for: Sperm selection with density gradient centrifugation and swim up: effect on DNA fragmentation in viable spermatozoa
Source: Sci Rep. 2019 May 16;9:7492. doi: 10.1038/s41598-019-43981-2 (PMC6522556; doi:10.1038/s41598-019-43981-2)
Supplement: Supplementary file 1 — Supplemental Information [file 41598_2019_43981_MOESM1_ESM.pdf]

Sperm selection with density gradient centrifugation and swim up: effect on DNA fragmentation in viable spermatozoa

M Muratori, N Tarozzi, F Carpentiero, S Danti, F M Perrone, M Cambi, A Casini, C Azzari, L Boni M Maggi, A Borini, E Baldi

Supplemental Table S1. Sperm parameters in subjects increasing and decreasing total sDF after selection with DGC and swim up (in the SW-G and SW-P group). Data are mean±SD.

	DGC			SW-G			SW-P		
**Variable**	**Sample Increasing** n=10	**Sample Decreasing** n=10	**p #**	**Sample Increasing** n=3	**Sample Decreasing** n=17	**p #**	**Sample Increasing** n=5	**Sample Decreasing** n=15	**p #**
Sperm Concentration (millions/ml)	75.5±45.2	55.60±32.0	0.271	74.9±56.6	148.3±110.2	0.281	33.07±39.83	99.50±52.22	0.012
Sperm Number (millions/ejaculate)	204.6±178.3	226.40±152.8	0.773	237.4±119.2	491.3±342.8	0.229	110.27±110.43	372.44±258.63	0.029
Progressive Motility (%)									
Before selection	47.7±14.0	43.6±11.8	0.487	63.0±8.9	53.4±9.0	0.102	52.17±4.36	52.64±12.74	0.931
After selection	76.1±9.3	81.5±5.9	0.137	76.5±4.9	85.9±14.9	0.405	75.33±13.43	83.43±6.06	0.074
P-value§	0.001	0.001		0.197	0.001		0.011	0.001	
Motility improvement	28.7±13.6	37.7±14.9	0.177	15.5±16.9	33.2±11.1	0.057	19.4±12.5	31.5±12.8	0.081

Sample increasing vs sample decreasing, t-Test, independent data. § before vs after selection, t-Test, paired data

Supplemental Table II. Sperm parameters in samples increasing and decreasing viable sDF after selection with DGC and swim up (in the SW-G and SW-P group). Data are mean±SD.

	DGC			SW-G			SW-P		
**Variable**	**Sample Increasing** n=12	**Sample Decreasing** n=8	**p #**	**Sample Increasing** n=8	**Sample Decreasing** n=12	**p #**	**Sample Increasing** n=8	**Sample Decreasing** n=12	**p #**
Sperm Concentration (millions/ml)	58.7±35.1	70.08±42.9	0.544	102.1±48.9	160.8±128.3	0.235	53.8±63.6	96.7±47.4	0.100
Sperm Number (millions/ejaculate)	218.6±172.3	213.42±162.6	0.946	286.1±142.3	564.6±376.5	0.063	197.5±235.9	358.0±253.3	0.171
Progressive Motility (%)									
Before selection	42.0±12.5	48.1±12.8	0.309	58.6±7.2	52.3±10.1	0.141	48.0±10.8	55.5±10.0	0.130
After selection	80.0±5.4	78.0±9.5	0.599	85.0±12.3	84.4±16.4	0.942	77.0±11.7	83.7±6.6	0.120
P-value§	0.001	0.001		0.004	0.001		0.002	0.001	
Motility Improvement	30.8±13.6	36.7±16.5	0.392	27.4±15.8	33.6±10.1	0.353	29.0±17.6	28.1±10.8	0.897

Sample increasing vs samples decreasing, t-Test, independent data. § before vs after selection, t-Test, paired data

Supplemental Table III. Multivariate analysis: total and viable sDF values after selection, before and after adjustment for basal (total or viable) sDF values (model 1) and for basal (total or viable) sDF and sperm progressive motility and number (model 2).

	**DGC** (mean±SE)	**SW-G** (mean±SE)	**SW-P** (mean±SE)
Total sDF before selection	41.48± 4.37	41.48±4.37	41.48±4.37
Total sDF after selection			
Unadjusted	47.44±5.93	26.99±5.93	31.42±5.93
Model 1	47.45±5.75	26.99±5.75	31.43±5.75
Model 2	44.29±5.92	30.97±5.91	30.60±5.60
Viable sDF before selection	26.07±4.45	24.85±4.45	20.45±4.45
Viable sDF after selection			
Unadjusted	39.78±5.91	23.60±5.91	21.70±5.91
Model 1	39.17± 5.85	23.31±5.84	22.60±5.87
Model 2	36.47±6.03	27.02±6.01	21.59±5.72

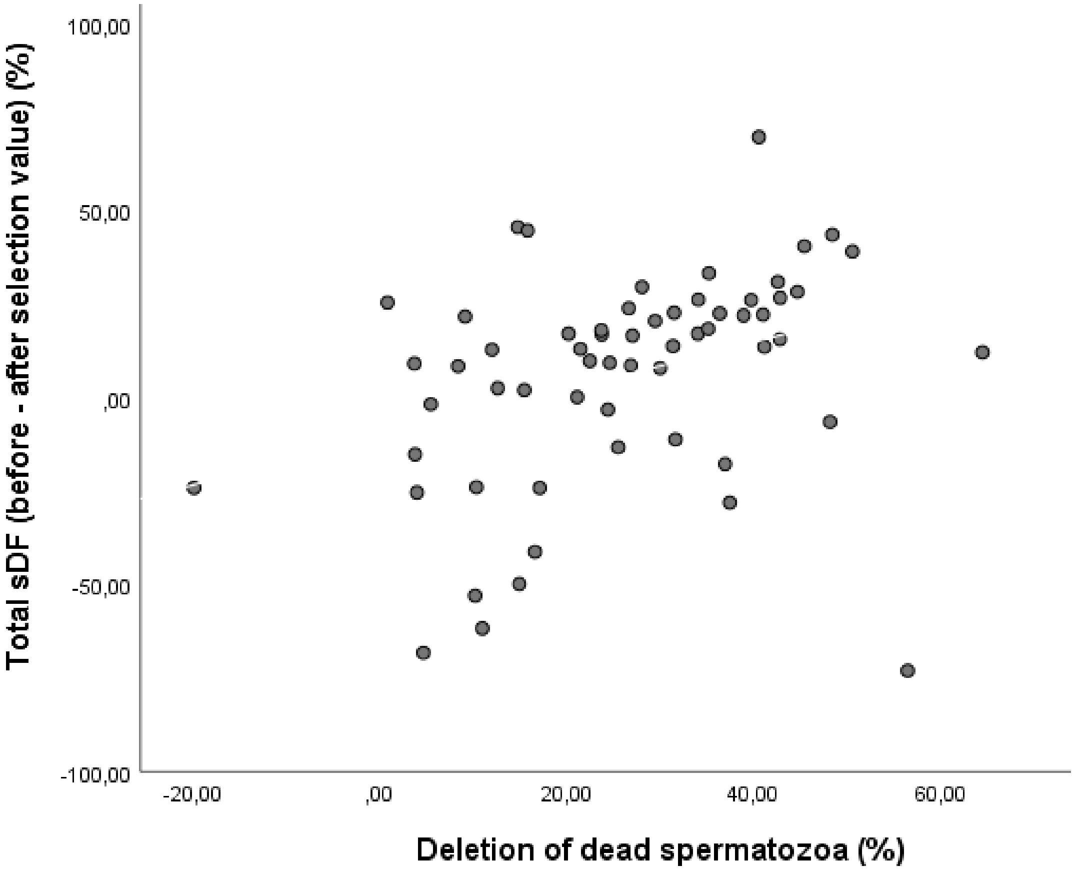

Supplemental Figure 1. Effect of elimination of dead spermatozoa during selection on the variation of total sDF. Difference of total sDF before and after selection plotted against the amount of dead spermatozoa eliminated during selection with DGC and swim up (n=60).
